# Evaluation of Maternal Nutrition Effects in the Lifelong Performance of Male Beef Cattle Offspring

**DOI:** 10.3390/vetsci10070443

**Published:** 2023-07-07

**Authors:** Roberta Cavalcante Cracco, Isabela Modolo Ruy, Guilherme Henrique Gebim Polizel, Arícia Christofaro Fernandes, Édison Furlan, Geovana Camila Baldin, Gianluca Elmi Chagas Santos, Miguel Henrique de Almeida Santana

**Affiliations:** Department of Animal Science, College of Animal Science and Food Engineering—USP, Av. Duque de Caxias Norte, 225, Pirassununga 13635-900, SP, Brazil; roberta.cracco@usp.br (R.C.C.); isabela.ruy@outlook.com (I.M.R.); guilhermepolizel875@gmail.com (G.H.G.P.); aricia.fernandes@usp.br (A.C.F.); edisonfurlan@usp.br (É.F.); geovana_baldin@usp.br (G.C.B.); gianluca1555@usp.br (G.E.C.S.)

**Keywords:** carcass traits, growth, maternal nutrition, Nellore

## Abstract

**Simple Summary:**

This study examined the effects of different prenatal nutrition treatments on pregnant cows and their calves. The cows were divided into three groups: NP (no supplementation), PP (supplementation during the last three months of pregnancy), and FP (supplementation throughout pregnancy). After calving, all cows and calves received the same environmental and nutritional conditions. Measurements such as body weight, body condition score, ribeye area, backfat thickness, and rump fat thickness were taken at various stages. The results showed some differences in cow weight, fat thickness, and body condition during pregnancy and postpartum. However, the effects of prenatal nutrition on the calves’ weaning weight and growth rates were minimal. Overall, the study concluded that prenatal nutrition had a limited impact on postnatal performance in beef cattle.

**Abstract:**

This study aimed to evaluate the effects of different prenatal nutrition treatments on pregnant cows and their progeny. One hundred and twenty-six pregnant Nellore cows (455.3 ± 8.1 kg) were allocated in three different nutritional treatments during pregnancy: NP—control, PP—protein-energy supplementation in the last 3 months of pregnancy, and FP—the same supplementation throughout pregnancy. After parturition, all cows and calves received the same environmental and nutrition condition. The body condition score (BCS), body weight (BW), ribeye area (REA), backfat thickness (BFT), and rumpfat thickness (RFT) were collected on four occasions during pregnancy in the cows and from birth to finishing in calves. All data (cows and calves) were submitted to an analysis of variance (*p* < 0.05) using a linear model (MIXED procedure; SAS software). The BW, RFT, and BCS from the cows showed significant differences in the middle third of pregnancy and pre-delivery and RFT postpartum (*p* < 0.05). For the offspring, the weaning weight showed a tendency (NP lighter than others). In terms of gain, the PP group tended to be higher in RFT at calving (*p* = 0.06), in REA at finishing (*p* = 0.09), and in ADG in the same period (*p* = 0.09). The prenatal nutrition strategies had little or no effect on the beef cattle postnatal performance.

## 1. Introduction

Maternal nutritional status is of great importance during pregnancy, as stimuli or insults during this period can lead to phenotypic and metabolic changes in the developing individual [1,2,3]. During embryo formation, the partition of nutrients from the mother prioritizes vital organs, like the heart, brain, and kidneys, and with this, tissues such as skeletal muscle and adipose, two of the main constituents of meat, are susceptible to the availability of nutrients offered [4,5]. Particularly, beef cattle, when raised on pasture, are more susceptible to nutritional insults due to seasonal changes in forage quality.

Thus, prenatal nutrition is of great importance in the meat industry, since its impacts can influence the animal’s physiology in several aspects [6]. In cattle, primary myogenesis begins within the first two months after conception, while secondary myogenesis occurs between the second and seventh or eighth months of gestation [7], it being an exclusive event of fetal life and not occurring after that period. Few muscle fibers are formed in primary myogenesis, and thus, maternal nutrition in early development has negligible effects on skeletal muscle development in this period. However, the vast majority of muscle fibers are formed during the second myogenesis, and a decrease in the number of fibers formed has irreversible effects on the progeny [5,8].

Adipose tissue, in turn, is formed primarily during fetal life and early postnatal life, ending its development in puberty [9]. The onset of adipogenesis occurs concomitantly with the second myogenesis, but the greatest formation of adipocytes occurs between late pregnancy and weaning [8]. That said, maternal nutrition during these developmental windows will affect the development of adipose tissue [10].

Related to this, several studies have already shown the effects of maternal nutrition on muscle and adipose tissue development [11,12,13,14,15] as well as on body weight (BW) [16,17] and other traits of economic importance [18,19,20]. However, the data found in the literature on maternal nutrition affecting the body performance of the progeny are sparse, evidencing the need for more studies in the area.

In this study, it was hypothesized that different protein-energy supplementation strategies during pregnancy in beef cattle alter the growth performance of their progeny. Thus, the objective of this study was to evaluate whether the different nutritional plans produced impacts on the muscular, adipose, and body development of the animals throughout their life, from the calving period to the finishing in feedlot.

## 2. Material and Methods

### 2.1. Ethics Statement

This study was approved by the Research Ethics Committee of the Faculty of Food Engineering and Animal Sciences from the University of São Paulo under protocol No. 1843241117, according to the National Council for the Control of Animal Experimentation guidelines.

### 2.2. Experimental Design

A total of 150 Nellore cows were fixed-time-artificially-inseminated (FTAI) with the semen of four bulls. At day 30 after FTAI pregnancy, diagnosis occurred (126 pregnant cows), and the animals were separated into three treatments: NP (control)—Not Programmed, without protein-energy supplementation; PP—Partial Programmed, protein-energy supplementation in the final third of pregnancy; and FP—Full Programmed, protein-energy supplementation during all of the gestation. Protein-energy supplementation was at the level of 0.3% BW. All groups received 0.03% BW mineral supplementation, included in protein-energy supplementation. More details about the groups and supplementation can be found in Schalch Jr. et al. [21].

Cows were blocked based on their age, BW, and body condition score and kept in pasture paddocks of *Brachiaria brizantha* cv. Marandu with access to the supplement and water ad libitum. After calving, the protein-energy supplementation was discontinued, and all the animals were kept together until the weaning stage, which occurred when they reached an average age of 220 days. The offspring received identical sanitary, vaccination, and feeding procedures, which were previously established on the Campus farm. Following weaning, the animals were segregated by their sex, without consideration for the previous treatments (NP, PP and FP), and were placed in distinct pastures. They continued to reside in their respective pastures throughout the rearing period. A total of 63 male offspring were used in this study (NP = 22, PP = 20, and FP = 21). More details about the rearing phase can be found in Polizel et al. [11].

At 19 months, young bulls initiated the finishing phase in the feedlot system lasting 106 days (15 of the adaptation period, and 91 of the effective feedlot). During this period, they received three different diets: an adaptation diet provided in the first 15 days (Dry matter (DM) = 48.1%; TDN = 71.0%; CP = 15.0%; NDF = 36.5%; Fat = 3.2%; Dry matter intake (DMI) = 2.21% of BW); a second diet during 35 days (DM = 53.6%; TDN = 73.6%; CP = 14.0%; NDF = 31.1%; Fat = 3.4%; DMI = 2.20% of BW); and a third diet for 56 days (DM = 60.6%; TDN = 76.2%; CP = 13.0%; NDF = 25.8%; Fat = 3.7%; DMI = 2.04% of BW).

**Figure 1:**
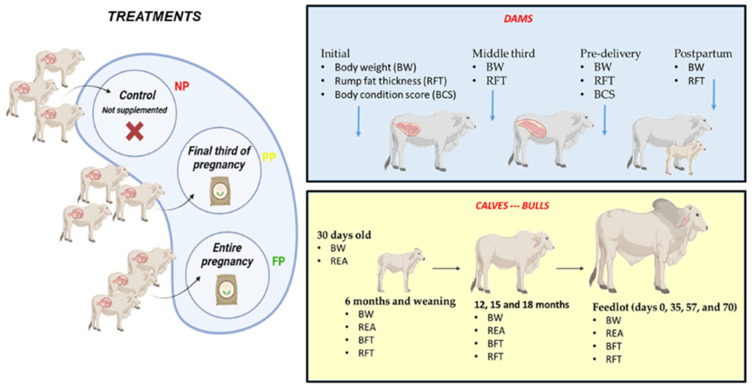
Experimental design of the three different prenatal nutrition strategies (NP, PP, and FP) and the collections carried out during the experimental period.

Figure 1 elucidates the experimental design in detail.

### 2.3. Ultrassound Evaluation

Ultrasound measurements of the ribeye area (REA), backfat thickness (BFT), and rump fat thickness (RFT) were conducted using an Aloka SSD-500 ultrasound machine. The ultrasound device was equipped with a 17 cm linear transducer operating at a frequency of 3.5 MHz, manufactured by Aloka Co., Ltd. (Wallingford, CT, USA). To ensure proper contact between the transducer and the animals’ skin, vegetable oil was utilized as a coupling agent during the measurements. Images in sections of the *Longissimus thoracis* muscle, between the 12th and 13th ribs, were taken to measure REA and BFT, while the RFT was measured by positioning the transducer in the final portion of the ileum, between the junction of the *Biceps femoris* and the *Gluteus medius* muscle [22]. The images were captured using the Lince software and later analyzed by a certified technician. Data were collected throughout the pregnancy, calving, rearing, and finishing phases. For the purpose of this study, data were collected at 30 days old (only REA), 6 months, weaning, 12 months, 15 months, 18 months, and the feedlot (days 0, 35, 57, and 70).

Regarding the ribeye area gain (REAg), backfat thickness gain (BFTg), and rump fat thickness gain (RFTg), the differences were calculated individually and inside each period, i.e., the gain in the rearing phase was obtained by subtracting the last measure collected in the rearing phase from the last measured in calving.

### 2.4. Weighting, Average Daily Gain, and Body Condition Score

Weights were obtained using an electronic scale from Coimma (Coimma Scales, Dracena, São Paulo State, Brazil) coupled to the cattle crush, where, regularly from birth to slaughter, the animals had their weight recorded. To obtain the average daily gain (ADG), a linear regression between age in days and weight was performed individually for the calving, rearing, and finishing phases. The body condition score (BCS) from the cows was performed in the FTAI and pre-delivery phases (initial and final, respectively) on a scale with values from one to nine, according to Richards et al. [23].

### 2.5. Statistical Analysis

All analyses were conducted using the MIXED procedure of the statistical package SAS^®^ version 9.4 (SAS Institute Inc., Cary, NC, USA). To assess the normality of the residuals obtained from the data, the Shapiro–Wilk test was applied, utilizing the UNIVARIATE procedure. In cases where the measurements deviated from normality, a logarithmic transformation (i.e., ln(trait + 1)) was employed. The homoscedasticity of residuals for principal effects was tested in the groups using Levene’s test. To evaluate the effects of treatments on phenotypes, analysis of variance was used, and the means were compared by the Tukey–Kramer test, with differences between treatments considered significant when *p* ≤ 0.05. The ages of the animal, cow, and sire were also considered in the linear model. Concerning the repeated measures, the same variables as those of the linear model were considered, and the time of data collection was also included. The covariance structure for the residuals was tested for each variable and chosen based on the Akaike information criterion (AIC). The variables REA, REAg, BFT, BFTg, RFTg, and weight used an unstructured analytic (un), while ADG used a first-order factor analytic (fa(1)). For this analysis, the model was as follows:

(1) $$y_{\mathrm{ijkl}}=\mu +\beta_{1}{\mathrm{Age}}_{m_{l}}+{\mathrm{Sire}}_{i}+{\mathrm{Treat}}_{j}+{\mathrm{Time}}_{k}+{\left(\mathrm{Treat}\times \mathrm{Time}\right)}_{\mathrm{jk}}+e_{\mathrm{ijkl}}$$

where $y_{\mathrm{ijkl}}$ is the observed variable from the lth animal, son of the ith sire, recorded on the jth treatment at the kth time of measurement (weaning, 12, 15, 18, and/or 22 months of age); μ is just a constant; $\beta_{1}$ is the regression coefficient of the covariate mother’s age; ${\mathrm{Age}}_{m_{l}}$ is the observed value for the mother’s age of the lth animal; ${\mathrm{Sire}}_{i}$ is the fixed effect of the ith sire; ${\mathrm{Treat}}_{j}$ is the fixed effect of the jth treatment; ${\mathrm{Time}}_{k}$ is the fixed effect of the kth time of measurement; ${\left(\mathrm{Treat}\times \mathrm{Time}\right)}_{\mathrm{jk}}$ is the fixed interaction between the treatment and time; and $e_{\mathrm{ijkl}}$ is the residual random term, which was assumed to be normally distributed with a covariance structure, as presented above. It must be noticed that, when the analysis was performed within time, this effect (as well as the treatment by time interaction) was removed from the model.

## 3. Results

### 3.1. Phenotypic Traits of Dams

The initial BW did not show any significant difference (Table 1). However, the BW in the middle third of gestation and the pre-delivery showed a significant difference (*p* < 0.01), with BW values in the FP group higher than in the others. Postpartum BW had no difference between the groups (*p* = 0.13). There were no differences in RFT in the early and middle thirds of gestation (Table 1), and pre-partum FP presented a higher RFT than the other treatments (*p* < 0.01). In the postpartum period, FP showed a higher RFT than NP (*p* = 0.04); however, PP did not show any difference from the others. The initial BCS was not different between treatments (*p* = 0.34; Table 1). In the pre-delivery period, the FP obtained a higher BCS than the NP (*p* = 0.04), and the PP group did not show any difference between the other two treatments.

### 3.2. Calves Performance

Relating to weight, there was a trend of difference at weaning (*p* = 0.08), where the NP group tended to be different from the others, and in the other periods, all treatments were similar (*p* > 0.05). When analyzing repeated measures over time, a trend in time x treatment interaction was shown (*p* = 0.07). In terms of ADG, a trend in the difference between treatments was identified for the finishing phase (*p* = 0.09), but there were no impacts of supplementation kind over time or any other period (*p* > 0.05; Table 2).

### 3.3. Ultrassound Carcass Traits

The REA was similar between the treatments both within and over time, with no significant differences being found (*p* > 0.05). When the REAg was analyzed, there was a trend for difference at the finishing phase (*p* = 0.09); however, no differences appeared at other periods or when it was carried over time (Table 3).

**Figure 2:**
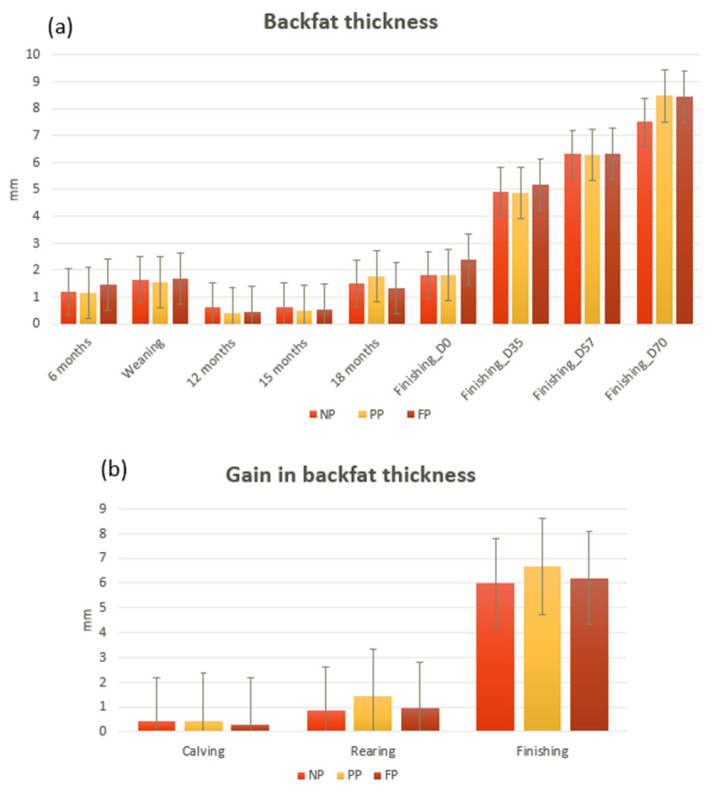
Lifelong backfat thickness of Nellore young bulls submitted to different prenatal nutrition strategies. The data are presented as the mean values of the traits ± the standard error of the mean. NP refers to the group without protein-energy supplementation, PP represents the Protein-Energy Last Trimester group (0.3% body weight protein-energy supplementation during the final third of pregnancy), and FP indicates the Protein-Energy Whole Gestation group (0.3% body weight protein-energy supplementation from pregnancy confirmation onwards). (**a**) represents each collection from calving to the finishing phase, and (**b**) represents the gain in each production phase. No statistical differences or trends were found in BFT (*p* > 0.1).

No differences between treatments were found for backfat deposition (*p* > 0.05) in all periods evaluated, for repeated measures over time, and for BFTg, showing a BFT similar for all three groups in a lifelong period (Figure 2).

All treatments had similar RFT when the data were analyzed within time (*p* > 0.05). When the analysis was carried over time, a trend in time x treatment interaction appeared (*p* = 0.06), i.e., the effect of time may depend on which treatment animals received. Relating to RFTg, there was a trend at the cow-calf phase (*p* = 0.06), where the PP group tended to be different from NP and FP and over time (*p* = 0.09), but at the rearing phase and feedlot, all treatments were similar (Table 4).

## 4. Discussion

This work followed the performance throughout the life of 63 Nellore bulls that underwent different prenatal nutrition approaches. During the 22 months of collection (age at slaughter), no significant differences were found between treatments (even though it was demonstrated that there were phenotypic differences between cows during the gestational period), but trends towards difference appeared at interesting points evaluated, indicating the presence of processes that can be studied further.

Stalker et al. [24] and Warner et al. [25] found differences in performance between cows that received prenatal supplementation and those that did not, similar to the results for animals in the FP group in our study, which increased BW, BCS, and RFT throughout pregnancy in relation to the other treatments. According to Melo [26], supplementation during pregnancy can increase cow BW, but with no effect in the postpartum period. However, regarding RFT, the postpartum analysis showed that the FP cows managed to maintain an energy reserve higher than that of NP cows, thus favoring the growth, maintenance, reproduction, and suckling of primiparous dams [27] and possibly shortening the interval between deliveries [28]. Differences observed in BCS and RFT can positively affect fetal development and the reproductive capacity of dams for the following breeding season and have long-term effects on the offspring [29], especially in tropical regions.

Some recent studies have evaluated the effects of energy-protein supplementation in pregnant Nellore cows [30,31,32,33]; however, due to the large number of variables (supplementation period and level, pasture quality, and other environmental characteristics) that may influence animal responses, the results show inconsistent effects.

During pregnancy, the nutrients ingested by the mother and sent to the fetus prioritize the formation of vital organs, such as the heart, brain, and kidneys [5], leaving tissues considered secondary, such as skeletal muscle, at the mercy of the availability of nutrients [4]. Thus, it is expected that maternal energy restriction causes changes in the muscle development of the offspring, as some studies on sheep [34] and cattle [13,35,36,37] have already demonstrated. Although there are indications of the impact of fetal programming on muscle, other studies, similarly to ours, did not observe differences in the REA [38,39] when the mothers underwent energy restriction. It is possible that this absence of differences in REA throughout life is due to a compensatory gain after birth.

Following this idea, we also measured the gains that occurred between the periods in an attempt to detect if there was a compensatory gain in postnatal life. A trend towards a difference in REAg during confinement was identified, where the PP treatment, which received protein-energy supplementation only in the final third, had a lower increase in REA compared to the others. Despite being only a trend in the treatment effect, important questions can be raised for future investigations.

The FP group may have received good levels of nutrition during their fetal formation and thus managed to maintain satisfactory muscle development. The NP group, which was considered our control and a good representative of beef cattle that undergo an extensive system in tropical regions, tolerates maintaining the same levels of muscle development as the FP since they are zebu animals, a naturally more rustic species, and perhaps, after generations of developing in Brazilian production, it has adapted and is able to overcome the adversities of this system. Still following this thought, and taking into account the primary window of muscle fiber formation that occurs only from the first third to the middle of the second third of pregnancy [5], it is possible to question that the supplementation received by the PP only in the final third of gestation was not able to interfere with the constitution of muscle fibers given the formation window, and adding to this, it may have annulled any previous adaptation of the species due to the “thrifty” phenotype [40].

The similarity between treatments for REA was also observed by Mohrhauser et al. [41] and Ramírez et al. [42]; however, differences in BFT measurements were reported in these studies. Results involving fat thickness and fetal programming are well dispersed in the literature. There are studies that reported an increase in thickness in the groups that underwent restriction, such as the two presented above, which probably happen due to a compensatory growth in adipose tissue [8]. However, other studies did not observe differences in fat thickness but observed differences in REA [12,37,43]. At the same time, other works, such as this one, did not find differences in any of the two characteristics, as in Wilson et al. [44] and Mulliniks et al. [45].

There was no difference in BFT, but some trends emerged in RFT and its gain. There was a trend in the interaction time x treatment, and when analyzing the RFTg, there was a trend in the calving period, where the PP group was superior to the others, and also over time. This trend towards a greater gain in fat thickness for the PP, coupled with the increase observed in some studies in groups that underwent restriction, reinforces the idea presented for the treatments previously when we talked about REAg.

Regarding weight, we observed a trend at weaning, where the control group had a lower weight compared to the others; however, no other trend was observed throughout life. In some studies, where the cows passed the pregnancy to pasture but received protein supplementation, the calves did not show differences in weight at birth; however, at weaning, there was an increase in weight when compared to the non-supplemented group [24,46,47]. Additionally, similar results were observed by Marques et al. [48], where cows that had an increase in the body conditioning score during the second and final thirds of gestation weaned heavier animals.

Although there were no differences for ADG between treatments, a trend was observed in the feedlot, where the PP group tended to have a weight increase above the others. When searching for studies in the literature, once again, scattered results were found. Some studies suggest there is no difference between treatments for ADG in the feedlot [43,44], while in Mulliniks et al. [45], animals whose mothers were able to maintain or gain weight during late pregnancy had higher ADG in the feedlot.

Given the results found in this work and in a search of the literature, it is possible to perceive the need for further studies on the effects of prenatal nutrition, it being the restriction or supplementation of cows. Still, some of our results tended to differ, which may become significant if the experiment is repeated and the conditions of the extensive system cause a more severe restriction in animals that do not receive protein-energy supplementation.

## 5. Conclusions

Maternal energy protein supplementation had little or no effect on the animal’s postnatal growth performance for the traits evaluated. However, we found some trends toward the difference between treatments in the characteristics (REA, RFT, and BW), suggesting that the three different prenatal nutrition strategies (NP, PP, and FP) may discreetly affect interest productive traits. The large number of phenotypic results brought by this article during the complete production cycle of beef cattle contribute to clarifying the long-term effects on male bovine progeny.

## Figures and Tables

**Table 1:** Average of body weight, rump fat thickness, and body condition score of Nellore cows submitted to different nutrition strategies, with their respective *p*-values.

Traits	NP	PP	FP	*p*-Value
**Body weight (kg)**				
Initial	461 ± 6.90	451 ± 9.38	454 ± 8.76	0.85
Middle third	490 ± 4.86 ^a^	493 ± 5.65 ^a^	516 ± 6.45 ^b^	<0.01 *
Pre-delivery	508 ± 7.23 ^a^	524 ± 9.07 ^a^	541 ± 10.1 ^b^	<0.01 *
Postpartum	503 ± 3.97	502 ± 5.21	518 ± 5.48	0.13
**Rump fat thickness (mm)**				
Initial	4.28 ± 0.61	4.31 ± 0.61	4.33 ± 0.61	0.92
Middle third	6.33 ± 0.40 ^a^	6.87 ± 0.38 ^a^	9.35 ± 0.50 ^b^	<0.01 *
Pre-delivery	7.23 ± 0.66 ^a^	9.24 ± 0.67 ^a^	12.5 ± 0.98 ^b^	<0.01 *
Postpartum	9.77 ± 0.47 ^a^	11.4 ± 0.48 ^ab^	12.6 ± 0.57 ^b^	0.04 *
**Body condition score**				
Initial	4.5 ± 0.09	4.6 ± 0.12	4.5 ± 0.09	0.34
Pre-delivery	5.4 ± 0.13 ^a^	5.6 ± 0.13 ^ab^	5.9 ± 0.13 ^b^	0.04 *

The data are presented as the mean values of the traits ± standard error of the mean. The lowercase superscript letters denote significant contrasts. NP indicates the group without protein-energy supplementation, PP represents the Protein-Energy Last Trimester group (0.3% body weight protein-energy supplementation during the final third of pregnancy), and FP indicates the Protein-Energy Whole Gestation group (0.3% body weight protein-energy supplementation from pregnancy confirmation onwards). * Statistical differences found between the treatments.

**Table 2:** Lifelong weight and weight gain of Nellore young bulls under different prenatal nutrition strategies.

Trait	Time	NP	PP	FP	*p*-Value ^1^	*p*-Value ^2^
**Weight (Kg)**	30 days	68.42 ± 2.71	68.81 ± 2.20	73.28 ± 2.89	0.35	0.30
	6 months	190.22 ± 4.92	199.09 ± 3.79	198.99 ± 5.08	0.27	
	Weaning	216.61^b^ ± 5.20	231.84 ^a^ ± 5.01	232.9 ^a^ ± 4.62	0.08 *	
	12 months	293.75 ± 5.03	296.88 ± 5.95	301.70 ± 5.54	0.46	
	15 months	370.01 ± 5.43	370.78 ± 6.02	382.99 ± 5.48	0.12	
	18 months	430.70 ± 4.79	429.78 ± 6.12	439.92 ± 4.34	0.13	
	Finishing_D0	452.07 ± 6.02	448.99 ± 7.17	464.01 ± 7.20	0.16	
	Finishing_D35	512.01 ± 6.73	518.97 ± 8.11	528.27 ± 8.04	0.21	
	Finishing_D57	556.52 ± 7.04	568.70 ± 9.29	568.94 ± 9.55	0.34	
	Finishing_D70	576.25 ± 8.11	590.92 ± 10.01	587.02 ± 10.19	0.39	
**ADG**	Cow-calf	0.85 ± 0.020	0.91 ± 0.017	0.92 ± 0.015	0.27	0.21
	Rearing	0.58 ± 0.011	0.56 ± 0.011	0.60 ± 0.013	0.34	
	Finishing	1.70 ^b^ ± 0.034	1.87 ^a^ ± 0.048	1.71 ^b^ ± 0.042	0.09 *	

The data are presented as mean values of the traits ± the standard error of the mean. Superscript lowercase letters (a, b) represent significant contrasts between treatments. *p*-values are indicated for comparisons between groups at the same age ^1^ and for repeated measures over time ^2^. NP refers to the group without protein-energy supplementation, PP represents the Protein-Energy Last Trimester group (0.3% body weight protein-energy supplementation during the final third of pregnancy), and FP indicates the Protein-Energy Whole Gestation group (0.3% body weight protein-energy supplementation from pregnancy confirmation onwards). * Trends to present difference between the treatments.

**Table 3:** Ribeye area throughout the life of Nellore young bulls submitted to different maternal nutrition strategies.

Trait	Time	NP	PP	FP	*p*-Value ^1^	*p*-Value ^2^
**REA (cm^2^)**	30 days	19.4 ± 0.9	19.85 ± 0.64	20.95 ± 1.01	0.78	0.62
	6 months	39.95 ± 1.32	43.41 ± 1.08	44.60 ± 1.16	0.35	
	Weaning	45.22 ± 1.03	47.51 ± 1.11	47.21 ± 0.94	0.62	
	12 months	58.78 ± 1.02	57.40 ± 1.05	59.32 ± 1.11	0.75	
	15 months	68.1 ± 1.08	67.99 ± 0.95	68.05 ± 0.89	0.96	
	18 months	77.18 ± 1.09	78.67 ± 1.01	78.60 ± 1.15	0.72	
	Finishing_D0	84.45 ± 1.22	86.90 ± 0.75	84.71 ± 1.06	0.35	
	Finishing _D35	92.75 ± 1.21	91.30 ± 1.16	94.89 ± 1.20	0.33	
	Finishing _D57	97.78 ± 1.22	102.33 ± 1.35	101.95 ± 1.24	0.31	
	Finishing _D70	97.50 ± 1.01	98.76 ± 1.22	98.26 ± 0.98	0.77	
**REAg**	Cow-calf	26.01 ± 1.41	27.20 ± 1.20	25.71 ± 1.17	0.88	0.81
	Rearing	18.21 ± 0.82	20.88 ± 1.39	19.94 ± 0.88	0.45	
	Finishing	13.52 ^a^ ± 0.85	11.01 ^b^ ± 1.12	13.10 ^a^ ± 1.01	0.09 *	

The data are presented as the mean values of the traits ± the standard error of the mean. Superscript lowercase letters (a, b) represent significant contrasts between treatments. *p*-values are indicated for comparisons between groups at the same age ^1^ and for repeated measures over time ^2^. NP refers to the group without protein-energy supplementation, PP represents the Protein-Energy Last Trimester group (0.3% body weight protein-energy supplementation during the final third of pregnancy), and FP indicates the Protein-Energy Whole Gestation group (0.3% body weight protein-energy supplementation from pregnancy confirmation onwards). * Trends to present difference between the treatments.

**Table 4:** Rump fat thickness throughout the life of Nellore young bulls under different maternal nutrition strategies.

Trait	Time	NP	PP	FP	*p*-Value ^1^	*p*-Value ^2^
**RFT (mm)**	6 months	2.23 ± 0.15	2.15 ± 0.18	2.50 ± 0.16	0.66	0.59
	Weaning	2.36 ± 0.17	2.84 ± 0.15	2.45 ± 0.20	0.39	
	12 months	1.04 ± 0.14	0.82 ± 0.16	1.02 ± 0.13	0.44	
	15 months	1.47 ± 0.16	1.56 ± 0.17	1.89 ± 0.13	0.46	
	18 months	2.94 ± 0.17	3.18 ± 0.19	3.29 ± 0.19	0.56	
	Finishing_D0	3.41 ± 0.15	3.14 ± 0.23	3.6 ± 0.21	0.65	
	Finishing _D35	6.10 ± 0.19	6.35 ± 0.27	6.79 ± 0.22	0.43	
	Finishing _D57	7.41 ± 0.20	7.06 ± 0.31	7.65 ± 0.25	0.40	
	Finishing _D70	8.51 ± 0.27	9.19 ± 0.39	9.72 ± 0.23	0.49	
**RFTg**	Cow-calf	0.09 ^b^ ± 0.14	0.69 ^a^ ± 0.15	0.0 ^b^ ± 0.17	0.06 *	0.09 *
	Rearing	1.90 ± 0.19	2.35 ± 0.17	2.29 ± 0.20	0.27	
	Finishing	5.20 ± 0.17	6.09 ± 0.32	6.10 ± 0.24	0.35	

The data are presented as the mean values of the traits ± the standard error of the mean. Superscript lowercase letters (a, b) represent significant contrasts between treatments. *p*-values are indicated for comparisons between groups at the same age ^1^ and for repeated measures over time ^2^. NP refers to the group without protein-energy supplementation, PP represents the Protein-Energy Last Trimester group (0.3% body weight protein-energy supplementation during the final third of pregnancy), and FP indicates the Protein-Energy Whole Gestation group (0.3% body weight protein-energy supplementation from pregnancy confirmation onwards). * Trends to present difference between the treatments.

## Data Availability

Not applicable.
